# Boosting of Thalamic D2 Dopaminergic Transmission: A Potential Strategy for Drug-Seeking Attenuation

**DOI:** 10.1523/ENEURO.0378-17.2017

**Published:** 2017-12-21

**Authors:** Fabricio H. Do-Monte, Gilbert J. Kirouac

**Affiliations:** 1 McGovern Medical School, The University of Texas Health Science Center, 6431 Fannin Street, Room 7.166, Houston, TX 77030; 2 Departments of Oral Biology and Psychiatry, Colleges of Dentistry and Medicine, Rady Faculty of Health Sciences, University of Manitoba Winnipeg, Manitoba R3E 0W2, Canada

**Keywords:** cocaine, dopamine, food seeking, nucleus accumbens, paraventricular thalamus, prefrontal cortex

## Abstract

This commentary focuses on novel findings by Clark et al. (2017) published in *eNeuro*, which show that dopamine D2 receptors (D2Rs) in the paraventricular nucleus of the thalamus (PVT) are involved in cocaine sensitization. We extend the discussion on how their findings contribute to our understanding of the role of the PVT in drug seeking by providing new insight on the role of the PVT in the regulation of food-seeking and fear responses. We also consider the significance of the neuroanatomical findings reported by Clark et al., that the PVT is reciprocally connected with areas of the brain involved in addiction and discuss the implications associated with the source and type of dopaminergic fibers innervating this area of the thalamus.

## Significance Statement

This commentary focuses on novel findings by Clark et al.(2017) published in *eNeuro* that dopamine D2 receptors (D2Rs) in the paraventricular nucleus of the thalamus (PVT) are involved in cocaine sensitization. The article describes experiments using a virally driven strategy in which the authors investigate the anatomic, physiologic, and behavioral properties of D2R expressing neurons in the PVT. Here, we will extend the discussion on how their findings contribute to our understanding of the role of the PVT in drug-seeking behavior by providing new insight into the recently described participation of this region in the regulation of food-seeking and fear responses. In addition, we will consider the significance of the neuroanatomical findings reported by Clark et al.(2017), that the PVT is reciprocally connected with areas of the brain involved in addiction and discuss the implications associated with the source and type of dopaminergic fibers innervating this area of the thalamus.

There is an emerging agreement that the paraventricular nucleus of the midline thalamus (PVT) is an important component of the forebrain circuits that mediate emotional and motivated behaviors (Kirouac, 2015). This has been in part driven by the observation that the PVT sends dense projections to the key areas of the brain involved in the modulation of such behaviors including the nucleus accumbens, prefrontal cortex, bed nucleus of the stria terminalis, and the central and basolateral nuclei of the amygdala (Li and Kirouac, 2008; Vertes and Hoover, 2008). Indeed, the recent surge of publications now establishes that the PVT contributes to the regulation of diverse biological responses including stress (Bhatnagar et al., 2003; Heydendael et al., 2011), fear (Li et al., 2014b; Do-Monte et al., 2015; Penzo et al., 2015; Choi and McNally, 2017), anxiety (Li et al., 2010; Heydendael et al., 2011), and food intake/sucrose-seeking responses (Choi et al., 2012; Haight et al., 2015; Labouèbe et al., 2016; Do-Monte et al., 2017; Livneh et al., 2017; Zhang and van den Pol, 2017). The PVT has also been a special focus of drug-seeking behavioral studies (Hamlin et al., 2009; James et al., 2010; Browning et al., 2014; Barson et al., 2015; Matzeu et al., 2015, 2016; Zhu et al., 2016) because of its remarkable diversity of neurotransmitters and neuromodulators linked to motivation (Kirouac, 2015; Lee et al., 2015). For example, a number of studies have provided experimental evidence that the neuropeptides orexins and cocaine- and amphetamine-related transcript (CART) in the PVT contribute to cocaine- and alcohol-seeking behavior (James et al., 2010; Barson et al., 2015; Matzeu et al., 2015, 2016). The PVT also contains dopamine fibers and receptors but how this neuromodulator critically linked to drug addiction contributes to PVT function remained unknown until now. A recent study by Clark et al.(2017) helps fill this gap by demonstrating that activation of dopamine D2 receptors (D2Rs) inhibited neurons in the PVT and that overexpression of these receptors attenuated locomotor sensitization to cocaine.

Using *in situ* hybridization for D2R mRNA in wild-type mice, Clark et al.(2017) first demonstrated that D2R are densely expressed in several subnuclei of the midline thalamus, with a particularly high expression in the PVT. This same pattern of expression was also observed in mice genetically modified to express green fluorescent protein (GFP) in D2R-containing neurons. Next, to determine the electrophysiological effects of D2R activation in PVT neurons, the authors performed whole-cell patch clamp recordings of PVT neurons *in vitro*. They found that a large proportion of D2R-containing PVT neurons were tonically active and that application of the D2R agonist quinpirole inhibited the firing rate of these cells without altering the response of non-D2R-containing neurons in the same region. Quantification of GFP positive neurons revealed that approximately two thirds of PVT neurons express D2R, a striking high density if we consider that only one third of the nucleus accumbens neurons express D2R (Richtand et al., 1995). High expression of inhibitory D2R in PVT neurons could serve to compensate for the lack of GABAergic inhibitory neurons in this region, as reported in previous studies (Celio, 1990) and confirmed here by Clark et al.(2017).

To characterize the role of D2R in drug-seeking behavior, the authors used an elegant viral approach to overexpress DR2 exclusively in PVT neurons, and consequently increase D2R signaling. Rodents repeatedly exposed to drugs of abuse become more hyperactive, a widely described phenomenon known as locomotor sensitization (Tirelli et al., 2003). Taking advantage of this robust behavioral alteration, the authors compared changes in locomotor activity in D2R-overexpressing mice versus control mice after repeated injections of cocaine (15 mg/kg, 5 d) or saline. Whereas both groups showed comparable levels of activity during the acquisition of locomotor sensitization, D2R-overexpressing mice exhibited a significant reduction in locomotion in response to a cocaine injection challenge performed a few days later. A role of D2R in the modulation of cocaine-seeking behavior has been previously described for the striatum (Dobbs et al., 2017). In addition, attenuation of cocaine-seeking responses following lesion or pharmacological manipulation of PVT has also been reported in previous studies (Young and Deutch, 1998; James et al., 2010; Browning et al., 2014; Matzeu et al., 2015, 2016). Here, Clark et al.(2017) linked these two observations by demonstrating for the first time that activation of D2R in PVT is sufficient to reduce locomotor sensitization to cocaine. Notably, no behavioral changes were detected when these same D2R-overexpressing mice were exposed to a set of other behavioral tests to evaluate sucrose-seeking, prepulse inhibition, anxiety, and fear responses. One possible explanation for the effects observed for locomotor sensitization and not the other behavioral tests is that overexpression of D2R in PVT neurons may not have been sufficient to detect the endogenous changes in dopamine levels occurring during these behavioral tests, but sufficient to detect the large increases in dopamine release associated with locomotor sensitization to cocaine administrations. Another possibility is that separate populations of neurons in the PVT mediate different behavioral responses. This is in part supported by the findings of Clark et al.(2017), as well as other evidence showing that PVT neurons are recruited to selectively modulate cocaine-seeking without affecting sucrose-seeking responses (Matzeu et al., 2015, 2017). Alternatively, it is also possible that the same PVT neurons could mediate both drug and palatable food-seeking rewards, but different inputs, systems of neurotransmission, or subtypes of receptors in these neurons are required for each one of these behaviors. While the latter explanation would be more consistent with the widely accepted view that drugs of abuse and palatable food share the same neural circuits in the brain (Volkow et al., 2013; Millan et al., 2017), further studies using optogenetic or chemogenetic tools to temporarily inactivate D2R-containing PVT neurons during these distinct behavioral tests may help to clarify this important issue.

To identify the brain regions innervated by D2R-containing PVT neurons, the authors infused a Cre-dependent adeno-associated virus (AAV-5-DIO-eYFP) into the PVT of DrD2-Cre mice to label the axonal projections of these neurons across the entire brain. The pattern of innervation observed after specifically targeting D2R-containing neurons was consistent with previous tracing studies describing the efferents of the posterior aspects of PVT (Li and Kirouac, 2008). A dense plexus of axonal fibers was found in the nucleus accumbens shell, bed nucleus of the stria terminalis, prelimbic prefrontal cortex, and agranular insular cortex. PVT fibers were also found in the lateral, basal, and central subnuclei of the amygdala, caudate putamen, and entorhinal cortex. It is also notable that D2R expression was restricted to the medial/posterior aspect of the PVT and largely absent in the anterior portion of PVT. Such neuroanatomical differentiation suggests that dopamine may exert a distinct effect on more posterior regions of PVT. Although some studies have demonstrated a functional dichotomy along the antero-posterior axis of PVT (Bhatnagar et al., 2003; Heydendael et al., 2011; Alamilla et al., 2015; Barson and Leibowitz, 2015; Barson et al., 2015; Do-Monte et al., 2017; Haight et al., 2017), a more detailed and systematic investigation of the role of these two subregions of the PVT in the modulation of learned and motivated behaviors is missing.

Clark et al.(2017) used a monosynaptic retrograde viral tracing method in DR2-Cre mice to identify the brain regions sending efferent projections to D2R-containing PVT neurons. Surprisingly, the authors described that some neurons in the nucleus accumbens and dorsal striatum make monosynaptic connections with D2R-containing neurons in the PVT and concluded that PVT is reciprocally connected with the striatum. This would be the first anatomic evidence that we are aware of demonstrating that neurons in the striatum send direct feedback projections to the thalamus. Previous studies using traditional retrograde tracer methods did not show the presence of striatal projections to PVT (Cornwall and Phillipson, 1988; Chen and Su, 1990; Li and Kirouac, 2012). Therefore, one should question whether the few labeled neurons that Clark et al.(2017) identified in the striatum were the result of some artifact produced by the pseudorabies viral tracing method used in their study (e.g., anterograde transsynaptic transfer as previously described by Zampieri et al., 2014). While it is possible that in some cases viral tracing methods may provide some advantages in terms of its sensitivity to reveal new brain pathways (Rajasethupathy et al., 2015), the existence of a direct striatal-thalamic projection will require confirmation using other tracing methods and a more accurate quantification of the number of labeled neurons in the striatum.

In contrast to the PVT-striatal pathway, the presence of reciprocal connections between the PVT and the prefrontal cortex reported in Clark et al.(2017) agrees with previous findings showing that the PVT receives feedback projections from the same prefrontal cortical areas that it innervates (Li and Kirouac, 2012). The type of information transmitted to the PVT by feedback projections from the prefrontal cortex is not known. Nonetheless, recent studies using optogenetics and neuronal recording methods have demonstrated a role of prelimbic cortical fibers to the PVT during the retrieval of both fear- and sucrose-predictive cues (Do-Monte et al., 2015; Otis et al., 2017). In addition, presentation of a food-predictive stimulus activated prelimbic neurons projecting to PVT (Haight et al., 2017); and lesions of PVT neurons attenuated goal-directed behavior and increased the incentive salience of reward-associated cues (Haight et al., 2015). Together, these findings suggest a role of corticothalamic projections in the regulation of emotional memories and behaviors. Whether this pathway is also involved in drug-seeking responses remains an open question. One reasonable interpretation of the experimental evidence collected thus far is that information from prefrontal cortical areas is integrated in the PVT along with ascending signals from the brainstem and hypothalamus in a way that contributes to the selection of appropriate behavioral responses (Kirouac, 2015; Do Monte et al., 2016).

It is also interesting to note that while the PVT receives projections from dopamine neurons in the hypothalamus and periaqueductal gray (Li et al., 2014a), the monosynaptic tracing method used by Clark et al.(2017) did not result in the colabeling of neurons in these regions with tyrosine hydroxylase, the rate limiting enzyme involved in the synthesis of dopamine. The reason for these negative results is likely due to the fact that transsynaptic uptake of the rabies virus by dopamine fibers may be inefficient because such fibers release their content extrasynaptically and do not make strong synapses (Wall et al., 2013). From a functional perspective, dopamine neurons in the hypothalamus and periaqueductal gray appear to relay arousal related information (Lu et al., 2006; Léger et al., 2010), and dopamine release in the PVT could serve to modulate the effects of cortical inputs to PVT neurons in a manner similar to how dopamine modulates cortical inputs to the striatum (Floresco, 2007).

Low levels of striatal dopamine D2R may predispose individuals to use stimulant drugs like cocaine (Dobbs et al., 2017). Consistent with this, evidence discussed in this article indicates that a large proportion of PVT neurons expressing D2R projects to the striatum, and that overexpression of these receptors attenuates the locomotor sensitization associated with cocaine administrations. These observations, along with previous findings showing that increased activity in the PVT is linked to locomotor sensitization and cocaine-seeking behavior (Young and Deutch, 1998; James et al., 2010; Browning et al., 2014; Matzeu et al., 2015, 2016), suggest that boosting D2R activity in the PVT may be a good strategy for the treatment of cocaine addiction. A pharmacotherapeutic approach to enhance D2R function based on the genetic makeup of each patient would represent one avenue for a more targeted treatment (Nielsen et al., 2014). Alternatively, the identification of a unique genetic marker in D2R-containing PVT neurons could be a logical step toward the development of new targeted therapies for substance abuse, similarly to what is being proposed for other neurologic disorders (Miyamoto et al., 2012; Chahrour et al., 2016).

## Synthesis

Reviewing Editor: Karen Szumlinski, University of California at Santa Barbara Decisions are customarily a result of the Reviewing Editor and the peer reviewers coming together and discussing their recommendations until a consensus is reached. When revisions are invited, a fact-based synthesis statement explaining their decision and outlining what is needed to prepare a revision will be listed below. The following reviewer(s) agreed to reveal their identity: Gavan McNally, Seema Bhatnagar

In consultation with two experts reviewers, we have reached the decision of “Revise-Editor Review Only”. This decision reflects the high quality of the commentary-both reviewers were very impressed by the scholarly nature of the piece and the skillfulness in placing with the findings of Clark et al. into the contemporary literature. However, both reviewers thought that key parts of the literature were omitted as highlighted below.

Reviewer 1:

The work by Flagel's group could perhaps receive more attention than it has. This work is very relevant to the role of PVT in motivated behaviour as well as locomotor activity, and hence to understanding many of the findings reported by Clark et al. A few more lines dedicated to this work would be very helpful to readers.

Reviewer 2:

1) on page 4, the authors discuss that Clark that some behavioral tests were significantly affected while others were not. They suggest that PVT neurons might selectively modulate cocaine-seeking but not sucrose-seeking but that this would be in contrast to the widely accepted view that drugs of abuse and palatable food share similar neural substrates. However, it is possible that the PVT could mediate both but that different inputs or outputs of the PVT are required for drug vs. palatable food-seeking rewards. So the D2 system may only be activated by one type of reward and another receptor system is involved in food reward- both could require the PVT and thus require similar circuits, just through different molecular pathways.

2)On page 5, the authors note that the medial/posterior PVT exhibts D2R but the anterior does not and go on to cite studies showing that the anterior-posterior gradient is functionally relevant but that this gradient has not been studied i leaned and motivated behaviors. However, extensive work has shown that the posterior PVT is critical for regulating stress responses, adaptations to repeated stress, circadian rhythms responsive to stress as well as some anxiety-related behaviors. This literature should be clearly cited.
